# DSCAMs: restoring balance to developmental forces

**DOI:** 10.3389/fnmol.2012.00086

**Published:** 2012-08-17

**Authors:** Andrew M. Garrett, Abigail L. D. Tadenev, Robert W. Burgess

**Affiliations:** The Jackson Laboratory, Bar HarborME, USA

**Keywords:** DSCAM, Dscaml1, cell adhesion, chemoattraction, retina, self-avoidance, chemorepulsion

## Abstract

Many of the models of neurodevelopmental processes such as cell migration, axon outgrowth, and dendrite arborization involve cell adhesion and chemoattraction as critical physical or mechanical aspects of the mechanism. However, the prevention of adhesion or attraction is under-appreciated as a necessary, active process that balances these forces, insuring that the correct cells are present and adhering in the correct place at the correct time. The phenomenon of not adhering is often viewed as the passive alternative to adhesion, and in some cases this may be true. However, it is becoming increasingly clear that active signaling pathways are involved in preventing adhesion. These provide a balancing force during development that prevents overly exuberant adhesion, which would otherwise disrupt normal cellular and tissue morphogenesis. The strength of chemoattractive signals may be similarly modulated. Recent studies, described here, suggest that Down Syndrome Cell Adhesion Molecule (DSCAM), and closely related proteins such as DSCAML1, may play an important developmental role as such balancers in multiple systems.

## Introduction

Despite its molecular classification as a cell adhesion molecule, Down Syndrome Cell Adhesion Molecule (DSCAM) is recognized for its physiological role in “self-avoidance” in Drosophila and mammals. Self-avoidance involves the repulsion of processes from the same cell during dendrite arborization or axon branching, and the prevention of fasciculation and clumping of cells of the same subtype during the development of structures with well-defined anatomies such as the mammalian retina. In this way, self-avoidance counteracts cell adhesion, which knits cells together. DSCAM is the best molecular entry point into the process of self-avoidance, which has been described, but little studied, for almost 30 years (Kramer and Stent, 1985; Kramer et al., 1985). Interestingly, recent work indicates that Drosophila Dscam1 may also be counteracting netrin-dependent chemoattraction, another well-characterized developmental process. Thus, Dscam is emerging as an active antagonist of cellular and molecular mechanisms that were previously viewed as acting largely unopposed. This view is unique because modulation was thought to be achieved simply by adjusting the strength of the positive signal and not by a distinct counteracting cue. In this review, we describe how this view has evolved as our understanding of Dscam's *in vivo* roles has grown.

## There are two sides to the force

Cell adhesion in many forms plays a critical role in development in general and in neurodevelopment in particular (Rutishauser and Jessell, 1988). Cells adhere to other cells and to the extracellular matrix during development through a variety of mechanisms including integrins, Ca^2+^-dependent cadherins, and Ca^2+^-independent cell adhesion molecules (Milner and Campbell, 2002; Gibson, 2011; Hirano and Takeichi, 2012). These adhesive interactions have been repeatedly shown to underlie many key aspects of development, including differentiation, cell migration, cell morphogenesis, and cell survival. An extreme view of cell adhesion as a motive force during development is put forward in the differential adhesion hypothesis (DAH). This idea proposes that the sorting of cells into different strata and tissues during development is similar to fluids separating based on differing miscibilities and surface tensions (Steinberg, 2007). Those cells that bind most tightly to each other form a dense core, and those that bind least tightly form the outermost layer, just as liquids separate based on surface tension. The forces in play are the attractive forces between cells and the tensions at the interfaces between two strata of differing adhesiveness. This model does a very good job of describing some developmental events such as epiboly, the expansion of cells over the yolk in some species during early development, but is probably not adequate to explain more complex cellular and tissue morphologies. Also, cells are active players in morphogenesis and are willing and able to expend energy to establish conformations that would not arise from passive processes. The elaborate dendritic and axonal arbors of neurons certainly challenge a simple model of morphogenesis.

**Figure 1:**
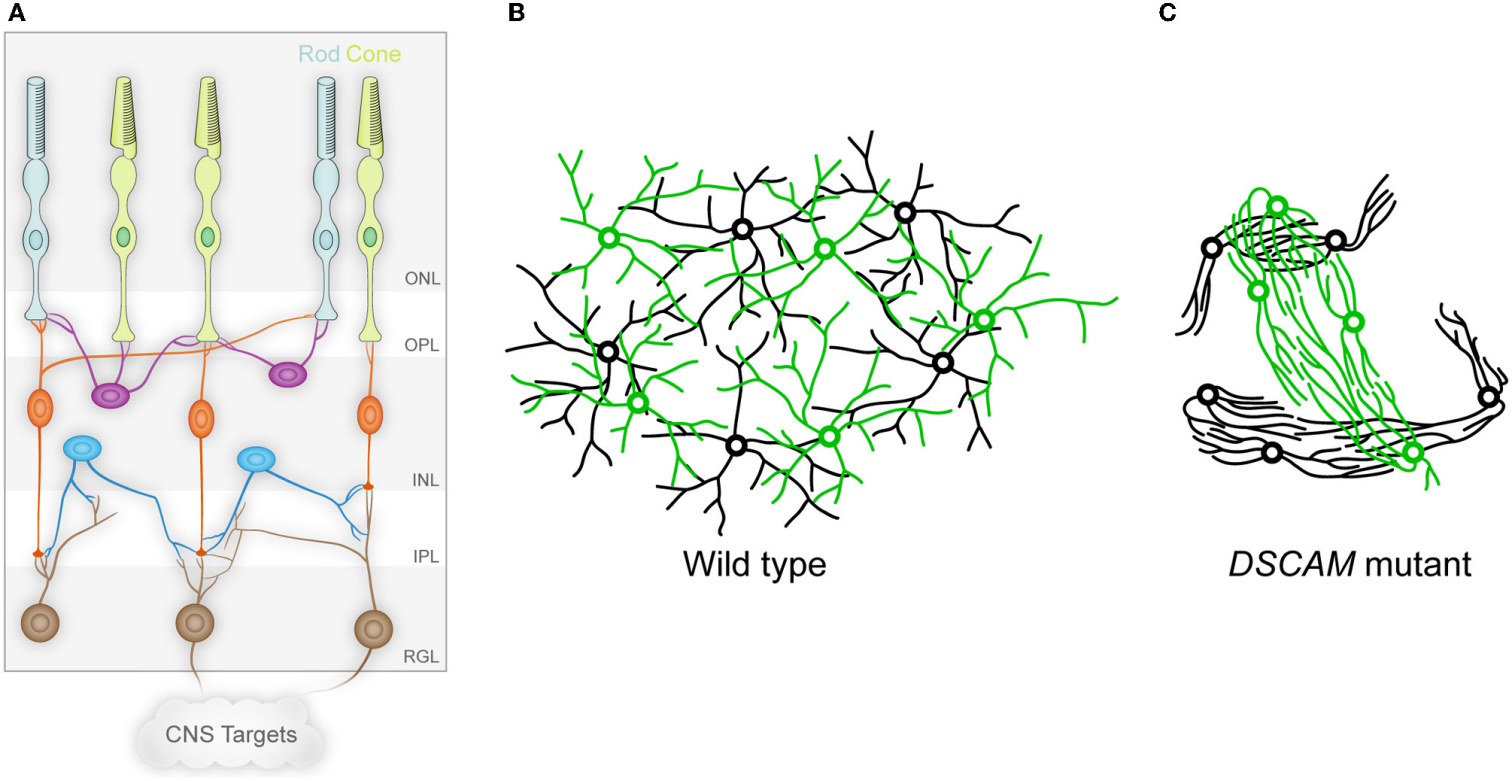
**The vertebrate retina and DSCAM in self-avoidance. (A)** The vertebrate retina is organized in columnar microcircuits connecting rod and cone photoreceptors in the outer nuclear layer (ONL) to horizontal (purple), bipolar (orange), and amacrine (blue) interneurons in the inner nuclear layer (INL) by synapses formed in the outer plexiform layer (OPL). The interneurons in turn form synapses in the inner plexiform layer (IPL) onto the dendrites of ganglion cells in the retinal ganglion cell layer (RGL), which project axons to the brain through the optic nerve. Establishing this anatomy involves both vertical organization into the different layers, and horizontal organization in which cells of a given type are non-randomly spaced and their dendritic arbors process information uniformly in circumferential space. **(B)** The circumferential spacing in a wild type retina is depicted for two cell types, in which the cell bodies of each cell type are non-randomly spaced from other cells of the same type, but are randomly distributed in relation to the cell bodies of other cell types. In addition, the dendritic arbors of these cells overlap, even within single cell types. This spacing is referred to as a mosaic. **(C)** In the absence of DSCAM, the dendrites and cell bodies of the neurons fasciculate and clump in a cell-type-specific manner, representing a loss of self-avoidance at the level of both individual cells and between cells of a given subtype. (A adapted from Garrett and Burgess, 2011.)

A more nuanced view of adhesion is put forward in an excellent review of retinal development (Galli-Resta et al., 2008). This review proposes that both the vertical, laminar structure of the retina, and the non-random, mosaic spacing of neuronal cell types in circumferential space of the retina can be explained by mechanical forces acting during development. To oversimplify the arguments made, the vertical organization arises because retinal ganglion cells adhere tightly to the extracellular matrix of the inner limiting membrane, whereas the adherens junctions of the outer limiting membrane knit the outermost cells of the retina together (Figure 1A). As cells proliferate in the space between, they occupy different strata based on their relative adhesiveness, much as one might predict from the DAH. These intermediate cells have also “let go” of both the innermost and outermost cells. The laminar and horizontal structures are further refined by adhesion between the processes of cells of a given type. In an analogy to a net, where the cell bodies are knotted nodes and the processes are the mesh, if the cells start in a heap, but are pulled as the eye grows, the result is a network of cells in a single layer that are evenly spaced from one another. This is a very attractive model that relies largely on adhesion between cells of a given type and mechanical forces of an expanding retina to achieve a final, highly organized anatomy. One interesting question that arises from this model is whether adhesion must be balanced by other forces. For example, do the cells of the inner nuclear layer (INL), the middle stratum of the retina, passively let go of the inner limiting membrane and ganglion cells, or are they kicked off? Similarly, as the heaped net of neurons is pulled outward, the cells of the heap have to be able to separate from one another, or the final anatomy looks like poorly cooked pasta. Thus, the adhesive forces between these cells allowing the network of processes to form must be counter balanced to prevent over-exuberant adhesion. This may occur with cell type specificity, since the pattern of each neuronal subpopulation in the retina arises largely independently, or even with subcellular specificity, allowing adhesion of distal processes, but preventing adhesion more proximally.

Work on the arborization of dendrites and patterning of neuronal territories in Drosophila and invertebrates such as the leech suggests that some neuronal subtypes “tile” through active repulsion, an opponent mechanism to adhesion. The processes of such cells occupy discrete, non-overlapping territories (hence the term tiling), and appear to actively repel neighbors from their space. When an axonal branch is ablated, for example, the vacated space is filled by the neighboring branch until they again abut without overlapping (Kramer and Stent, 1985). In Drosophila, some instances of tiling may reflect cell processes responding to a physical demarcation such as a segment boundary or the midline, which may have a specialized matrix or extracellular environment. However, neurons can be made to cross such boundaries by mutating genes such as the atypical cadherin Flamingo (Gao et al., 2000; Grueber et al., 2002). Therefore, the territories occupied by the processes of these cells are not necessarily constrained in their inherent physical size, but are limited by repellent interactions with their neighbors or their extracellular environment.

It is currently unclear if repulsion represents an extreme form of preventing adhesion, or if those mechanisms that counter balance adhesion are distinct from those that actually promote repulsion, although the possibility that adhesive mechanisms can be coopted for such purposes has been discussed (Cavallaro and Dejana, 2011). However, the relationship between repulsion and self-avoidance remains unanswered largely because neither mechanism is well understood.

Perhaps processes analogous to dendrite arborization are axon guidance and cell migration. It is clear that in these processes, both attractive and repellent signals are integrated to achieve complex effects such as attraction to and crossing of the midline, and doing so only a single time without turning back (Stein and Tessier-Lavigne, 2001). Interestingly, the same signal can be both attractive and repellent, depending on the receptors present on the receiving cell (Hong et al., 1999). In another example, the guidance of migrating inhibitory neurons from the ganglionic eminence during cortical development, SLIT proteins appear to direct migration through repulsion. However, some of this activity may be more accurately described as funneling migrating neurons to their destinations by creating a non-permissive environment for their migration (Zhu et al., 1999; Wichterle et al., 2003). Such differences border on semantics, but demonstrate mechanistic distinctions. Is an extrinsic guidance signal actively attractive or repellent, or is it simply more or less attractive to growth? With cell adhesion, the question could become whether cells not sticking is a passive failure to adhere, an active repulsion, or an active prevention of adhesion that simply renders cells indifferent to one another.

Interestingly, DSCAM may be a key player in both of these analogous developmental processes of cell adhesion and chemoattraction. DSCAM is a cell adhesion molecule in the immunoglobulin superfamily with one additional closely related vertebrate gene family member, Dscam-like1 (*Dscaml1*), and four Drosophila homologs, DSCAMs 1–4 (Yamakawa et al., 1998; Agarwala et al., 2001; Millard et al., 2007). DSCAMs (used here to refer collectively to all gene family members) bind specifically and homophilically in cell aggregation assays (Agarwala et al., 2000, 2001; Yamagata and Sanes, 2008). However, only some of their proposed functions in neurodevelopment are consistent with adhesion, and in other instances they appear to balance both chemoattraction and cell adhesion, and can even serve as a repellent, as described below.

Much of our knowledge of DSCAMs comes from Drosophila, in which *Dscam1* has the distinction of extensive molecular diversity that arises through alternative exon usage (Schmucker et al., 2000). In total, the Drosophila *Dscam1* gene can encode 19008 extracellular domains, and these bind homophilically with isoform specificity (Wojtowicz et al., 2004). Individual neurons express a stochastic handful of *Dscam1* isoforms on their cell surfaces and in this way can be uniquely identified, able to recognize “self”—other processes of the same cell—but remaining blind to the processes of neighboring cells (Neves et al., 2004). This self-recognition leads to self-avoidance, and two processes of a given cell end up repelling, promoting functions like dendrite arborization or axon branching within a single cell (isoneuronally) (Wang et al., 2002; Zhan et al., 2004; Hughes et al., 2007; Matthews et al., 2007; Soba et al., 2007). Thus, Dscam1-mediated self-avoidance prevents self-crossings within dendritic arbors, but allows overlap with neighboring neurons through its molecular diversity and the isoform specificity of the interactions.

The self-avoidance mechanism described above is contact dependent; two Dscam1-laden processes of the same cell must encounter one another to signal self-avoidance and repulsion through homophilic recognition. This process is much more efficient when the processes are constrained to a two-dimensional space and therefore more likely to encounter one another. In this function, Dscam1 function intersects with integrin-mediated adhesion to the extracellular matrix (Han et al., 2012; Kim et al., 2012). The developing dendrites of Drosophila dendrite arborization neurons (da neurons) exhibit Dscam1-dependent self-avoidance and grow largely in contact with the laminin rich ECM on the basal surface of the body wall epithelium through integrin-dependent adhesion to laminin. In the absence of integrins, these dendrites become “enclosed,” where segments of the dendrites become engulfed or surrounded by the epithelial cell. Under these conditions, the incidence of self-crossings within the dendritic arbor of an individual da neuron increases, but these are “non-contacting” self-crossings, where an enclosed dendrite segment crosses another dendrite, but the intervening epithelial cell prevents contact, and therefore, Dscam1-mediated self-avoidance. These studies clearly demonstrate that Dscam1's function is integrated with, and to some extent dependent upon, other cell adhesion systems such as integrins, but is Dscam1's repellent self-avoidance function balancing adhesion? Results from studies of the mammalian DSCAMs would suggest that they are indeed counteracting adhesion.

## Self-adhesion: you must learn to control your feelings

The function of DSCAMs has been studied in the mouse retina in both wild type mice and mice mutant for either *Dscam* or *Dscaml1* (Fuerst et al., 2008, 2009). In the mouse, *Dscam* is expressed in a subset of amacrine interneurons (dopaminergic amacrine cells (DA) and b-NOS-positive amacrine cells) and most retinal ganglion cells (Figure 1A). *Dscaml1* has a different, non-overlapping expression pattern, with expression in the rod circuit: rod photoreceptors, rod bipolar cells (RBCs), and AII amacrine cells. In the absence of *Dscam* or *Dscaml1*, the cells that would normally express the gene adhere abnormally and in a cell-type-specific manner (Figures 1B,C). Cells of a given type, DA cells for example, have fasciculated dendrites and the cell bodies are clumped together. Notably, while bNOS-positive amacrines exhibit a similar phenotype, these two cells types do not co-clump or co-fasciculate, despite the physical proximity of the DA and bNOS-positive cells. DSCAML1 mediates a similar self-avoidance function in RBC dendrites and AII amacrine cells. The defects in the *Dscam* mutant retina are consistent with homophilic cell-to-cell interactions based on studies of chimeric eyes (Fuerst et al., 2012).

Is this cell-type-specific fasciculation and clumping truly a failure of self-avoidance analogous to Drosophila Dscam1 function? It appears to be. Individual DA neurons examined in isolation before their dendrites overlap extensively with those of neighboring sister cells showed significantly more isoneuronal self-crossing in the *Dscam* mutant mice than in wild type controls (Fuerst et al., 2008). However, the *Dscam* mutant retina is also “vertically” disorganized: dendrites from particular cell types are no longer strictly confined to laminar strata within the plexiform layers. Perhaps the fasciculation observed in the *Dscam* mutant retina represents non-contacting crossings, such as one sees in Drosophila when the normal two-dimensional stratification of processes is disrupted even with intact Dscam1 function? One way to answer this question would be to investigate DSCAM function in a two-dimensional system, such as cultured neurons. If mutant neurons exhibit increased self-crossings when adherent to a culture dish, this would establish that DSCAM has a legitimate isoneuronal self-avoidance function, which may also extend to self-avoidance between cells of a given subtype.

An exciting implication of this cell-type-specific clumping and adhesion is that there is a cell-type-specific adhesion code that is unmasked by the loss of DSCAM, leading to the overly exuberant adhesion seen in the mutant retina (Fuerst and Burgess, 2009; Garrett and Burgess, 2011). The identities of the molecules that underlie this adhesion code remain unknown, but in this scenario, DSCAM-mediated self-avoidance is once again balancing adhesion. Whether this self-avoidance function truly involves repulsion or simply makes cells indifferent to one another remains to be determined in a vertebrate system. In support of the indifference model, *Dscam*-expressing cell types have heavily overlapping dendritic arbors, creating many self-type crossings, although as described above, careful examination in the *Z*-axis may show these to be non-contacting crossings. Similarly, developing processes of DA neurons do not show an interaction consistent with an actively repellent response (Keeley and Reese, 2010). Finally, the *Dscam* and *Dscaml1* genes are expressed in some abundant and closely packed cell types, including *Dscam* expression in most retinal ganglion cell types from a very early age, and *Dscaml1* expression in rod bipolar cells (Fuerst et al., 2009). It is hard to imagine how a cell in such a population could be actively repellent to all other cells in the population. Furthermore, such a repulsion mechanism could force some cells to leave the lamina in which they belong. In favor of a repellent mechanism are the results from Drosophila showing active repulsion (Montague and Friedlander, 1991; Matthews et al., 2007). Nonetheless, whether actively repellent or simply indifferent, mouse DSCAMs do seem to mediate self-avoidance both isoneuronally and between cells of a specific type. One interpretation of the clumping and fasciculation phenotype that is seen in the absence of the DSCAMs is that these proteins are serving to balance self-adhesion that otherwise runs unopposed.

In opposing self-adhesion, DSCAMs may allow mosaic spacing of cell bodies to occur without actively promoting it. Indeed, the non-random spacing of cells and the establishment of exclusion zones may depend on cells finding a homogeneous position in a gradient of a secreted signal. The MEGF10 and 11 proteins appear to be serving such a role in the retina, and alterations in their expression levels lead to alterations in the cell body spacing pattern at the interface between high and low expressing regions (Kay et al., 2012). The DSCAMs are not the only molecules that balance self-adhesion in the retina; there are several cell types that do not appear to express either *Dscam* or *DscamL1*. Indeed, horizontal cells mutant for *PlexinA4* exhibit increased dendritic self-crossings, although their arbors are not as severely disrupted as the affected cell types in *Dscam* mutants and the cell body spacing is maintained (Matsuoka et al., 2012).

## DSCAM in guidance: this is not the netrin you are looking for

Interestingly, DSCAMs serving such a balancing role may also be true in the analogous process of axon guidance. DSCAMs are proposed to function as netrin receptors (Ly et al., 2008; Liu et al., 2009). This is based on a similar domain structure to DCC and Neogenin (Unc41-family netrin receptors), as well as physical interactions with both DCC and netrin. In addition, knockdown of *Dscam* in filleted spinal cord preparations resulted in a defect in commissural axon guidance consistent with impaired netrin function. Furthermore, Drosophila *Dscam1* acts semi-redundantly with *Dscam3* to affect axon guidance in both netrin-dependent and –independent ways (Andrews et al., 2008). However, an examination of *Dscam* knockout mice failed to show netrin-like defects in axon guidance, suggesting DSCAM's interaction with netrin signaling might have more subtle effects (Palmesino et al., 2012).

A study of Drosophila dendrite arborization suggests that Dscam1's impact on netrin signaling may indeed be more complex, with Dscam1-mediated self-avoidance effectively balancing netrin-dependent attraction (Matthews and Grueber, 2011). In the absence of Dscam1, some of the dendrite arborization defects observed could be accounted for by dendrite attraction to sources of netrin. Ectopic expression of netrin could redirect the poorly arborized *Dscam1* mutant dendrites in an attractive manner. Finally, neurons mutant for both *Dscam1* and the netrin receptor Frazzled/DCC had disorganized dendritic arbors that were not attracted to the netrin source. Thus, Dscam-mediated self-avoidance of developing dendrites seems to promote the formation of a normal arbor by balancing the attractive cues provided by netrins. In other contexts, the loss of such a balancing factor could indeed cause an axon guidance phenotype that would have to be carefully interpreted to be sure it was not the result of overly exuberant attraction or repulsion.

It is also interesting to consider that DSCAM is proposed to function as a netrin receptor in axon guidance, and Dscam1 and -2 are involved in axonal branching and tiling in Drosophila, but axonal phenotypes are poorly studied in vertebrates (Wang et al., 2002; Millard et al., 2007). Some defects in targeting and ipsilateral/contralateral segregation of retinal ganglion cell axons within the lateral geniculate nucleus have been observed, but it remains to be determined if these effects are the result of DSCAM loss in the retina, the target, or both (Blank et al., 2011). Thus, Drosophila DSCAMs appear to impact netrin signaling for both axon and dendrite guidance, with the latter being a function of Dscam1-dependent self-avoidance balancing netrin-dependent attraction.

Do vertebrate DSCAMs function exclusively to balance adhesion or attraction in self-avoidance? Perhaps not, based on results from the chick retina (Yamagata and Sanes, 2008). In this system, Dscam and related adhesion molecules Dscam-like, Sidekick1, and Sidekick2 define distinct sublaminae in the synaptic inner plexiform layer (IPL). The knockdown of each of these proteins causes cells that normally stratify in that sublamina to mislocalize, and driving the ectopic expression of one of these proteins in a cell that does not normally express it redirects the dendrites to the lamina of the overexpressed protein. The most straightforward explanation of these data is that the adhesion molecules define the lamina in which neurons will arborize their processes and thus promote synaptic connectivity. As the IPL contains the presynaptic processes of amacrine and bipolar cells and the postsynaptic dendrites of retinal ganglion cells, this interaction is presumably a homophilic adhesion that promotes their co-stratification. Such a mechanism essentially fits the DAH, where the segregation of cell types, or in this case their processes, is determined by a selective or differential adhesiveness.

The extent to which the DSCAMs drive laminar specificity in the IPL of the mouse retina remains unclear. Studies of the first reported *Dscam* allele (*Dscam*^*del17*^) and the *Dscaml1* mutant mice suggest that laminar specificity is intact (Fuerst et al., 2008, 2009). Other studies in a second, spontaneous allele of *Dscam* did show a disruption of laminar specificity, but whether these differences are due to allele-specific effects or the differences in the genetic background of the mutant mouse strains also remains to be determined (Fuerst et al., 2010). Furthermore, the Semaphorin/Plexin signaling pathway mediates laminar specificity for at least some retinal cell types in the mouse (Matsuoka et al., 2011a,b).

## DSCAM intracellular signaling: i thought these things were complicated on the outside

How do DSCAMs mediate self-avoidance, and could the same molecules really be responsible for effects ranging from balancing netrin-dependent attraction and cell-type-specific adhesion to promoting laminar specificity through homophilic binding? It seems possible, although the molecular mechanisms are only just being explored.

Human DSCAM binds to P21 Activated Kinase1 (PAK1) through its juxtamembrane intracellular domain (Li and Guan, 2004). This same interaction is preserved in Drosophila, although in flies it is indirect and mediated by Dock, an SH2-SH3 adapter protein (Schmucker et al., 2000). PAK1 has many developmental roles, most notably being downstream of small GTPases and mediating actin cytoskeletal rearrangements. The role of PAK1 in DSCAM-mediated neurodevelopmental processes is unknown. Furthermore, it is unknown if PAK1 interacts with related molecules such as DSCAML1, which is divergent in sequence in the proximal intracellular domain. It is attractive to postulate that those functions that are shared between mammalian DSCAM and Drosophila Dscaml1 will use the same intracellular pathways, but this remains to be tested.

An interaction that does appear to be shared between DSCAM and DSCAML1, and also with more divergent family members such as Sidekick1 and Sidekick2, is an interaction of the C-termini of these proteins with multi-PDZ domain containing proteins such as MAGIs, PSD95, and Chapsyn110 (Yamagata and Sanes, 2010). This interaction was identified in a yeast-2-hybrid assay, and knockdown of MAGI2 in the chick retina perturbs Sidekick2 function in laminar specificity.

Interestingly, since these interacting proteins are multi-PDZ containing scaffolding molecules, the composition of these DSCAM-containing complexes may contribute to DSCAM's numerous possible functions. For example, in a complex with proteins specifying the cell-type-specific adhesion code, DSCAMs could serve to mask their function to balance adhesion. This could be through a physical inhibition of their extracellular adhesion properties, or through an inactivation of their intracellular signaling pathways. A similar interaction with components of the netrin signaling apparatus could also underlie DSCAM's role in balancing attraction; however, since DSCAM also directly interacts with DCC through an extracellular domain, complexing with other proteins scaffolded by multi-PDZ domain proteins may not be necessary. Furthermore, a change in the composition of the complex could easily allow DSCAMs to mediate self-avoidance early in development and to serve an adhesive function later in development, directing laminar specificity, such as in the chick retina, or synapse maturation, as suggested by DSCAML1 in the rod circuit in the mouse retina. Finally, it is a hypothetical possibility that DSCAMs could serve multiple roles simultaneously in a single cell if the complexes have different composition in different subcellular compartments.

A caveat to the idea that the composition of complexes scaffolded by multi-PDZ domain proteins confers specialized function to DSCAMs is the fact that Drosophila DSCAMs do not have an obvious PDZ-interacting C-terminus. Again, it is tempting to assume that conserved functions such as self-avoidance will happen through conserved intracellular signaling pathways, but this does not have to be the case, and as PAK1 demonstrates, the same pathways may be activated through direct or indirect interactions in one species or another. These ideas concerning signaling/adhesion complexes remain highly speculative, but are an active line of investigation.

## Summary

DSCAMs are important neurodevelopmental proteins conserved from flies to mammals. Much of their function appears to be to provide balance to better understood processes such as cell adhesion or chemoattraction. In their absence, there is overly exuberant adhesion and fasciculation between cells of specific subtypes in the mouse retina, and overly exuberant outgrowth of sensory dendrites toward sources of netrin in the fly body wall. These phenotypes demonstrate the importance of balancing these developmental mechanisms, and that this is sometimes an active process; not adhering is more than simply failing to adhere. DSCAMs serve additional roles, including potentially adhesive functions in examples such as the chick IPL. This diversity of activities may be dependent on interactions with other proteins in an adhesion/signaling complex, as suggested by DSCAMs' binding to multi-PDZ domain scaffolding proteins. Thus, while the diversity of DSCAM activities may rival its molecular diversity in Drosophila, the emerging commonality is that DSCAM balances competing forces.

### Conflict of interest statement

The authors declare that the research was conducted in the absence of any commercial or financial relationships that could be construed as a potential conflict of interest.
